# Neural Markers Predict Tendon Healing Outcomes in an Ovine Achilles Tendon Injury Model: Spontaneous Repair Versus Amniotic Epithelial Cell-Induced Regeneration

**DOI:** 10.3390/ijms26062445

**Published:** 2025-03-09

**Authors:** Valeria Giovanna Festinese, Melisa Faydaver, Delia Nardinocchi, Oriana Di Giacinto, Mohammad El Khatib, Annunziata Mauro, Maura Turriani, Angelo Canciello, Paolo Berardinelli, Valentina Russo, Barbara Barboni

**Affiliations:** 1 Unit of Basic and Applied Biosciences, Department of Biosciences, Agro-Food and Environmental Technologies, University of Teramo, 64100 Teramo, Italy; valeria.festinese@unicam.it (V.G.F.); mfaydaver@unite.it (M.F.); odigiacinto@unite.it (O.D.G.); melkhatib@unite.it (M.E.K.); amauro@unite.it (A.M.); mturriani@unite.it (M.T.); acanciello@unite.it (A.C.);; 2 School of Advanced Studies, Center for Neuroscience, University of Camerino, 62032 Camerino, Italy

**Keywords:** peripheral nervous system, Achilles tendon, amniotic epithelial stem cells, tendon innervation, neuropeptides, neural markers

## Abstract

Tendon injuries pose a clinical challenge due to tendons’ limited recovery. Emerging evidence points to the nervous system’s critical role in tendon healing, with neural markers NGF, NF-200, NPY, CGRP, and GAL modulating inflammation, cell proliferation, and extracellular matrix (ECM) remodeling. This study investigates the predictive role of selected neural markers in a validated ovine Achilles tendon injury model, comparing spatio-temporal expression patterns in regenerating tendons transplanted with amniotic epithelial stem cells (AECs) versus spontaneous healing (CTR) 14 and 28 days post-injury (p.i.). AEC-treated tissues showed a spatio-temporal modulation of NF-200, NGF, NPY, CGRP, GAL, and enhanced ECM remodeling, with greater cell alignment, lower angle deviation, and accelerated collagen maturation, with a favorable Collagen type 1 (COL1) to Collagen type 3 (COL3) ratio. Pearson’s matrix analysis revealed significant positive correlations between NGF, CGRP, and GAL expression, along a positive correlation between the three neural markers and cell alignment and angle deviation. As opposed to CTR, in AEC-treated tendons, lower levels of NGF, CGRP, and GAL correlated positively with improved tissue organization, suggesting these markers may predict successful tendon regeneration. The findings highlight the neuro-mediated activity of AECs in tendon regeneration, with NGF, CGRP, and GAL emerging as key predictive biomarkers for tendon healing.

## 1. Introduction

Achilles tendon injury (AT) is a common condition that affects athletes and non-athletes alike, with an estimated prevalence of AT of 2.35 per 1000 people-years in the general population [1]. It is more common among athletes (6%) compared to amateur exercisers (4%) [2]. Different factors, such as age [2] and specific sports (running, gymnastics) [2,3], contribute to a higher prevalence of AT. Tendon injuries pose significant clinical challenges due to the limited intrinsic healing capacity of tendons [4,5], with Achilles tendon injuries often resulting in a chronic condition that severely impact patients’ physical, psychological, financial, and well-being [6,7].

Current treatments for AT, including surgical options (autografts, allografts, xenografts), pharmacological approaches (non-steroid anti-inflammatory drugs, corticosteroids), and physical therapy, often have limited success [8,9,10,11]. This limited efficacy highlights the fact that tendon healing is a complex process. Persistent inflammation, the formation of scar tissue, hypervascularization, and improper extracellular matrix (ECM) remodeling are common factors hindering successful repair and often leading to the development of chronic tendon injuries [12]. Despite our current understanding of the systems involved, particularly as regards vascularization and immunity [13,14], many aspects remain unclear, including the contribution of the nervous system to tendon healing [15,16,17,18].

Recent research highlights the significant role of the nervous system in tendon homeostasis and healing, demonstrating a complex interplay with the blood and the immune systems [16,19,20,21]. Neural elements, including neurotrophic factors, neuro-mediators, and structural and enzymatic neuroproteins, have been shown to play crucial roles in orchestrating the healing of tendon tissues [20,22,23]. Under physiological conditions, the peripheral nervous system is primarily confined to the paratenon and endotenon of the tendon [16,19,20]. However, during the healing process [12], extensive nerve ingrowth occurs, with sensory and autonomic mediators regulating inflammation and regeneration in a time-dependent manner [22,24]. In this context, Nerve Growth Factor (NGF) and neurofilament (NF) proteins such as NF-200 are recognized to control cellular communication and promote angiogenesis and inflammatory responses during repair [20,23,24,25]. Analogously, neuropeptides such as neuropeptide Y (NPY), galanin (GAL), and calcitonin gene-related peptide (CGRP) exhibit pleiotropic effects during all phases of tendon repair, modulating inflammation, tenocyte proliferation, extracellular matrix (ECM) deposition, tissue architecture recovery, and nociception [20,23,24,26,27]. Disruptions in the expression of these neuro-mediators may contribute to sustained inflammation, ECM deregulation, and chronic pain [24,26,27,28,29], highlighting the need for innovative models to investigate effective regenerative strategies.

In this context, stem cell-based approaches offer a valuable tool to explore the neurobiological aspects of tendon healing, which is characterized by three distinct phases—inflammatory (about 7 days), proliferative (about one month), and the subsequent remodeling process [30]. Among stem cell sources, amniotic epithelial stem cells (AECs) are known to possess a pro-regenerative paracrine action and are able to teno-differentiate in vitro and in vivo [14,31,32,33]. AECs promote Achilles tendon healing through allo- and xenotransplantation due to their direct contribution to tissue regeneration and through paracrine modulation of the inflammation, immunity, and ECM remodeling [14,31,32,34,35]. Unlike tendons undergoing spontaneous repair—which exhibit delayed ECM remodeling, excessive collagen 3 (COL3) deposition [36], and a prolonged inflammatory response [14,31,34,35,37]—AEC-treated tendons demonstrate an accelerated regenerating process. By 14 days post-injury (p.i.) (proliferative phase), transplanted AECs modulate innate immunity, shifting the macrophage (Mϕ) balance from pro-inflammatory M1Mϕ to the anti-inflammatory M2Mϕ subpopulation [14,34,37]. At this stage, AEC-treated tendons show an early substitution of COL3 immature fibers with mature collagen 1 (COL1), the main ECM component in healthy tendons [14,31,37]. By 28 days p.i. (early remodeling phase), differently to spontaneous healing tissues, the ECM is almost reorganized in transplanted tendons [14,31,34,35,37], with COL1 alignment and reduced cellularity, indicating an accelerated ECM regeneration [14,31,37]. Indeed, Transforming growth factor- β1 (TGF-β1) modulation plays a key role in this process. In AEC-treated tendons, at 14 days, its upregulation supports ECM deposition by enhancing COL1 and COL3 synthesis [14,38], whereas by 28 days, its downregulation is observed, which aligns with tendon maturation and helps prevent excessive fibrotic response [14]. This modulation contributes to a favorable COL1/COL3 ratio, supporting ECM remodeling and structural integrity. Additionally, AEC-treated tendons exhibit a decrease in vascular density, with blood vessels aligning with the longitudinal axis of the tendon [14,31,35]. While the angiogenic response is essential in tendon healing, incomplete remodeling and regression of the vascular network eventually compromises tendon biomechanical properties [14,31,35]. These observations are coherent with an accelerated regeneration promoted by transplanted AECs compared to spontaneous repair [14,31].

Furthermore, evidence of a link between AECs and neural lineages [39,40,41,42,43,44] suggests that AECs might also be able to influence nerve-mediated regenerative processes. Specifically, AECs express neural markers and can differentiate into neuroectodermal lineages, a trait attributed to their common embryonic origin, the epiblast [39,40,45,46]. This shared lineage predisposes AECs to manifest neural characteristics and may be the base of their influence on the regeneration process. By producing critical neurotrophic factors and neuropeptides, AECs may actively modulate local inflammatory responses, enhancing tendon recovery [45,47,48]. A better understanding of nervous system involvement in tendon healing could be crucial in the identification of prognostic markers to distinguish between proper and defective p.i. recovery. In this perspective, the validated ovine experimental model of Achilles tendon injury hereby used provides a valuable translational tool: ovine tendons closely resemble human tendons in size, structure, and function [49,50], and the model exhibits spatio-temporal variations at both structural and molecular levels comparable to human patients [50,51]. AEC allotransplantation-induced regeneration of the Achilles tendon represents a solid protocol for an enhanced structural and functional recovery of the tendon, as opposed to spontaneous healing, generally resulting in a slower and defective process [14,31,34,35,37].

Leveraging the above-described AEC-induced tendon regeneration as opposed to spontaneous repair in sheep [14,31,34,35,37,52], this research initially focused on elucidating the distribution and expression patterns of the main neural markers, NF200, NGF, CGRP, NPY, and GAL, in these two healing conditions. In particular, comprehensive insights into the spatio-temporal dynamics of these molecules during the proliferative (14 p.i.) and early remodeling (28 days p.i.) stages [14,31,34,35,37] was assessed in both AEC-treated and spontaneous repair conditions. Additionally, this study examined whether the differential modulation of these neural markers between regenerating tissues and tendons undergoing spontaneous repair could allow us to identify potential predictive molecular patterns to determine healing outcomes. The findings address a key knowledge gap on the neuro-mediated role of AECs in tendon regeneration, with the nervous system and its related neural markers emerging as predictive molecules for tendon healing in a validated tendon regeneration model, which may hold prognostic value for the clinical management of tendon injuries.

## 2. Results

### 2.1. Amniotic Epithelial Cells Spontaneously Express Neural Markers Prior to Allotransplantation

A quantitative assessment on the percentage of AECs at P0 and P3 expressing NGF, GAL, CGRP, and NPY was conducted pre-transplantation in injured tendons.

**Figure 1:**
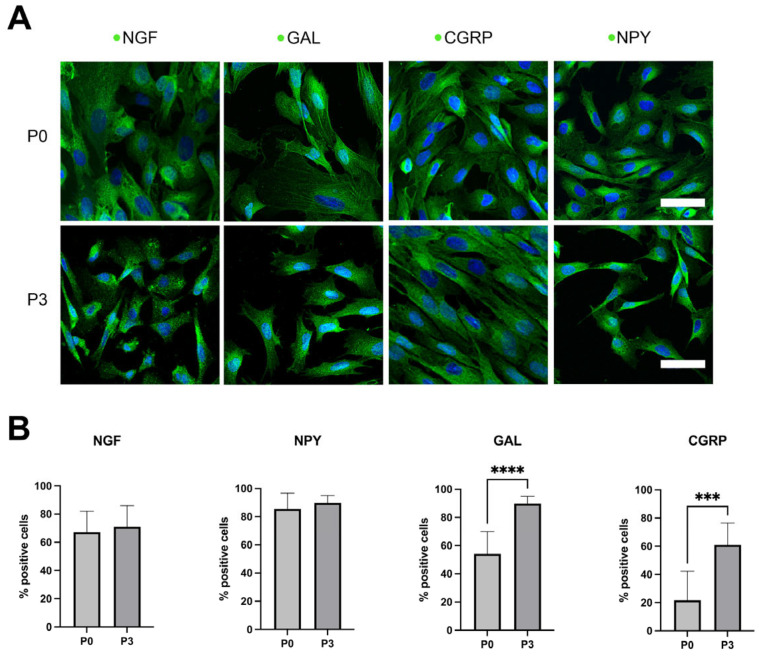
(**A**) Expression of neural markers NGF, NPY, CGRP, and GAL in AECs at P0 and P3, documented by ICC (green fluorescence). Nuclei were counterstained with DAPI (blue). Positivity for each marker was tested at both the first passage (P0, top) and third passage (P3, bottom). Scale bar: 50 μm. (**B**) Quantification of the percentage of cells positive for neural markers at both P0 and P3; a high percentage of positive cells was detected, with no significant difference among passages, in both NPY and NGF. A significant increase in expression at P3 with respect to P0 occurs in the case of GAL (*p* < 0.0001, ****) and CGRP (*p* < 0.001, ***), with both markers displaying a high expression level at P3.

ICC analysis and the quantification of the percentage of positive cells confirmed the expression of all tested molecules in AECs at both passages (Figure 1A). More specifically, more than 60% of the cells was always positive in the case of NPY and NGF at both P0 and P3, with no significant difference among passages. To the contrary, a significant increase in expression at P3, with respect to P0, was detected in the case of GAL (*p* < 0.0001) and CGRP (*p* < 0.001), with a high percentage of positive cells (CGRP: P0 = ~50%, P3 = ~90%; GAL: P1 = ~20%, P3 = ~50%) at P3 in both markers (Figure 1B).

### 2.2. AEC Culture Determines a Differential NGF Release

**Figure 2:**
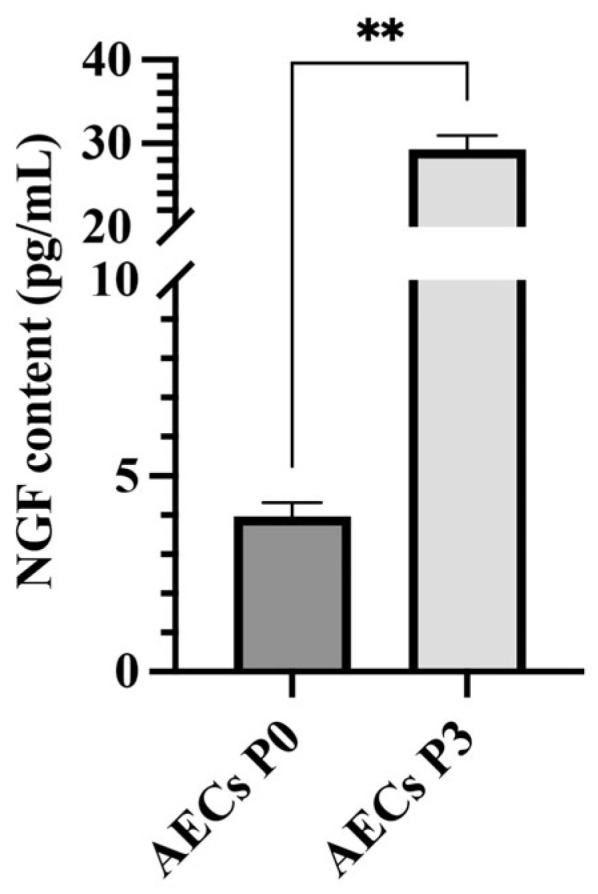
Levels of NGF secretion by AECs at P0 and P3 documented via ELISA. AECs tested significantly higher for NGF secretion at P3 with respect to P0 (*p* < 0.01, **).

The release profile of NGF content within AECs resulted to be passage-dependent, with significantly elevated values within AECs at P3. In particular, as shown in Figure 2, relevant levels of NGF were found in all AEC-derived conditioned media (CMs), independently from their passages (P0 3.97 ± 0.36 pg/mL for P0 and 29.27 ± 1.65 pg/mL for P3). Interestingly, CMs derived from AECs at P3 contained significantly higher levels of NGF, showing a nearly threefold increase, compared to those from P0 (*p* < 0.01, Figure 2).

### 2.3. Retrieval of PKH26-Labeled Cells Supports AECs’ Involvement in Neuro-Mediated Tendon Regeneration

**Figure 3:**
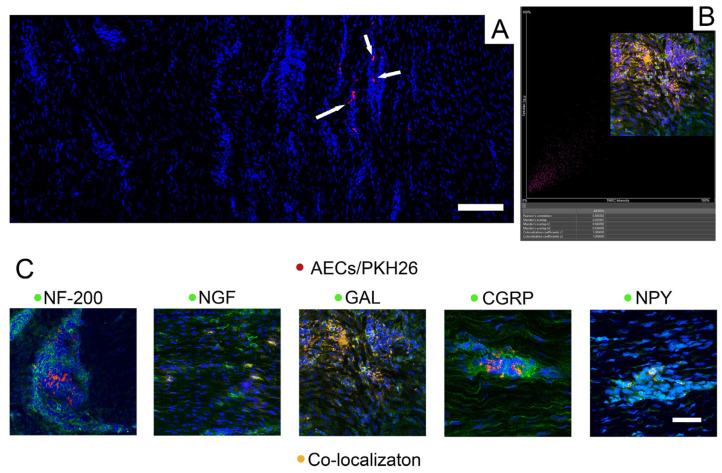
Retrieval of PKH26-labeled cells at 28 days p.i. supports AECs’ involvement in tendon regeneration. Twenty-eight days is hereby included as a reference time point for PKH26-positive cell (red fluorescence) retrieval. (**A**) An example of PKH26-positive cells (white arrows) localized within AEC-treated tendons. Scale bar: 200 μm. (**B**) An example of the co-localization analysis of selected ROI to which Pearson and Manders coefficients were applied to confirm the observed co-localizations; Pearson’s correlation coefficient (PCC) = 0.86; Mander’s overlap coefficients: 0.94 (M1 and M2). (**C**) No co-localization was observed in the case of NF-200 (green fluorescence), whereas co-localization of PKH26-labeled cells (red) with the remnant neural markers tested (green fluorescence) is observed as a yellow/orange color, given by the overlapping of green and red fluorescence signals. Nuclei were counterstained with DAPI (blue). Scale bar: 50 μm.

PKH26-labeled AECs were retrieved and analyzed for their co-localization with neural markers following transplantation at both time points (Figure 3: 28 days p.i.). PKH26-labeled cells were primarily observed in close proximity to the center of the lesion site (area 3) at both time points (Figure 3A). PKH26-labeled cells did not co-localize with NF-200, but neurofilaments were found to surround clusters of PKH26-positive cells (Figure 3C). Furthermore, IHC for the assessment of neural markers revealed that PKH26-labeled cells displayed co-localization for NGF, NPY, GAL, and CGRP at both time points (Figure 3B,C: 28 days p.i.).

### 2.4. Allotransplantation of AECs Modulates Neural Markers in an Ovine Achilles Tendon Injury Model

All samples were assessed for the considered neural markers NF-200, NGF, NPY, CGRP, and GAL to verify their distribution and localization within the analyzed tendons.

**Figure 4:**
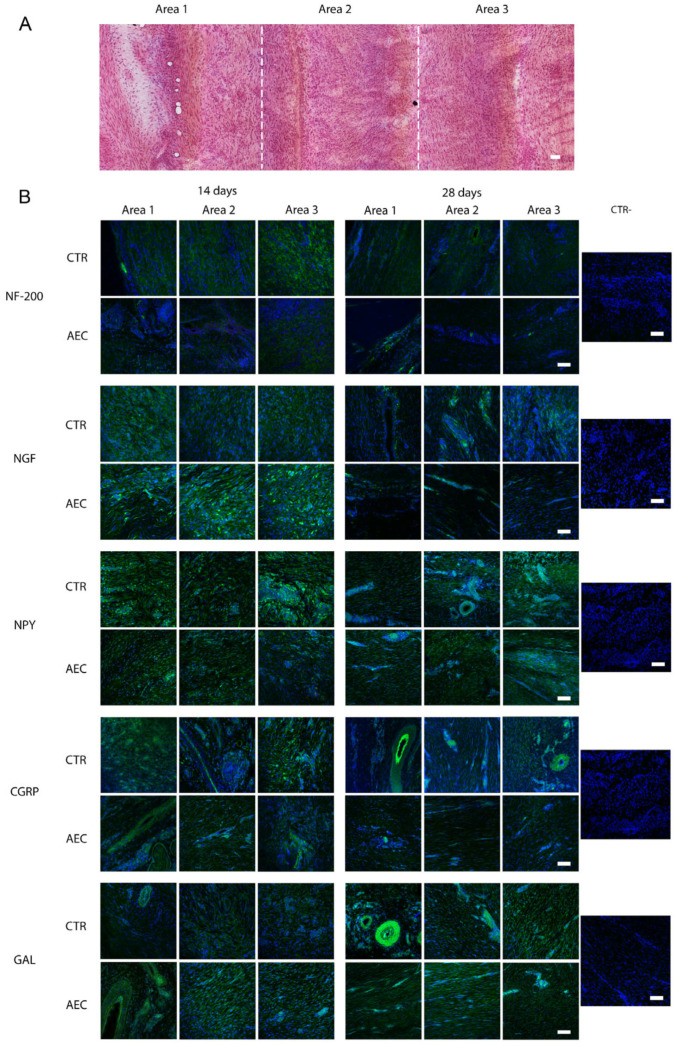
(**A**) An example of a hematoxylin/eosin-stained large image of the injured tendon (CTR) at 14 days showing the extension of the lesion and the localization of the considered areas. Scale bar: 100 μm. (**B**) Expression patterns of neural markers NF-200, NGF, NPY, CGRP, and GAL documented by immunohistochemistry (green fluorescence). Nuclei were counterstained with DAPI. In each panel, three areas are shown, with area 1 representing the peripheral region up to area 3 representing the center of the lesion. All analyzed markers show variations in expression patterns among the different experimental groups (AEC-treated tendons vs. CTR). On the right, negative controls (CTR-) for the immunofluorescence technique are shown. Scale bar: 100 μm.

The injured tendons (CTR and AEC-treated tissues) were divided in three consecutive areas starting from the outer area of the tendon (closer to the paratenon), area 1, up to the center of the lesion, area 3 (Figure 4A).

IHC assessment confirmed the low expression levels of the analyzed neural markers within the healthy tendon, with occurrence limited to the endotenon and to the area closer to the paratenon, and no positivity in the tendon proper as per previous reports in different species [53,54,55].

On the other hand, transplantation of AECs appeared to impact the distribution and modulation of the expression of neural markers.

In particular, nerve ingrowth was observed in the repairing tendons (CTR) and to a lesser extent in AEC-treated tendons. At 14 days p.i., samples from both experimental groups showed the presence of few nerve fibers in the outer area (area 1), closer to the paratenon, with the CTR group displaying an increased positivity towards the center of the lesion (area 3). At 28 days p.i., however, while, in CTR tendons, nerve endings were still evenly distributed across all areas, NF-200 positivity in AEC-treated tendons was mostly limited to the endotenon and the outer area confining with the paratenon, retracting from the tendon proper (Figure 4B).

As regards the other markers, NGF expression appeared to have the most widespread localization across all areas analyzed at day 14 in all experimental groups but with a higher expression for AEC-treated with respect to CTR tendons; such distribution was still evident at 28 days in CTR, where NGF was also localized near large blood vessels. Instead, in the AEC-treated group, NGF expression appeared to be only in the endotenon and close to the paratenon (Figure 4B).

For the analyzed neuropeptides, variations in the distribution pattern were observed in the AEC-treated group with respect to CTR group. In the CTR group, NPY expression on day 14 appeared to be present in all considered areas, whereas on day 28, it was higher in areas 2 and 3 of the tendon; these same areas on day 28 p.i. showed the presence of large blood vessels, characterized by greater NPY expression around such vases. To the contrary, a diffused pattern of expression was observed in the internal areas of the tendon (areas 2–3) in the AEC-treated group at both 14 and 28 days post-surgery (Figure 4B). Finally, CGRP and GAL showed a similar expression pattern distribution in the injured tendon. In the CTR group the expression of both markers appeared relatively constant over time; at 28 days, both markers were observed around large blood vessels (Figure 4B). To the contrary, the expression of both CGRP and GAL in the AEC-treated groups appeared to be only in the endotenon and close to the paratenon of the injured tendons at 28 days p.i. Interestingly, several host tissue cells both in CTR and AEC-treated tendons were positive in the cytoplasm for all analyzed neural markers (Figure 4B).

**Figure 5:**
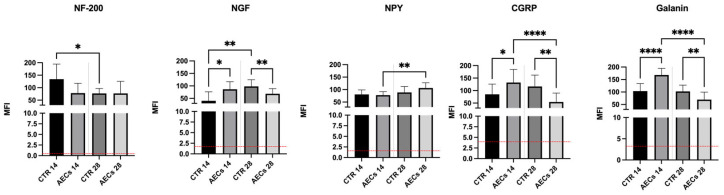
Quantification of mean fluorescence intensity (MFI) revealed a significant upregulation of the analyzed markers across all experimental groups compared to healthy tendons (red baseline). Significant CTR vs. AEC-treated tendon (AEC) variations in the expression of neural markers were observed, also showing a time dependency. Values were considered statistically significant for * *p* < 0.05, ** *p* < 0.01, **** *p* < 0.0001.

Quantification of the mean fluorescence intensity (MFI) revealed a significant upregulation of the analyzed markers across all experimental groups compared to healthy tendons (*p* < 0.0001). At 14 days post-transplantation, only CTR tendons exhibited a significant increase in nerve ingrowth to then decrease at 28 days post-injury (*p* < 0.05), while the expression of NF-200 consistently remained lower in the AEC-treated group throughout all time points (*p* < 0.05) (Figure 5). Moreover, while NGF expression increased over time in the CTR group (14 days vs. 28 days, *p* < 0.01), becoming significantly higher than the AEC group at 28 days (*p* < 0.01), it instead displayed a significant peak on day 14 in the AEC-treated group compared to CTR (*p* < 0.05) and remained constantly expressed over time (Figure 5). NPY exhibited a significant increase over time in the AEC-treated group (14 days vs. 28 days, *p* < 0.01); to the contrary, CGRP and GAL exhibited elevated levels observed at 14 days compared to CTR (*p* < 0.05), whereas levels decreased over time in the AEC-treated group (14 days vs. 28 days, *p* < 0.0001) (Figure 5).

### 2.5. AEC Allotransplantation Affects the Expression of Tendon-Related Gene Markers

**Figure 6:**
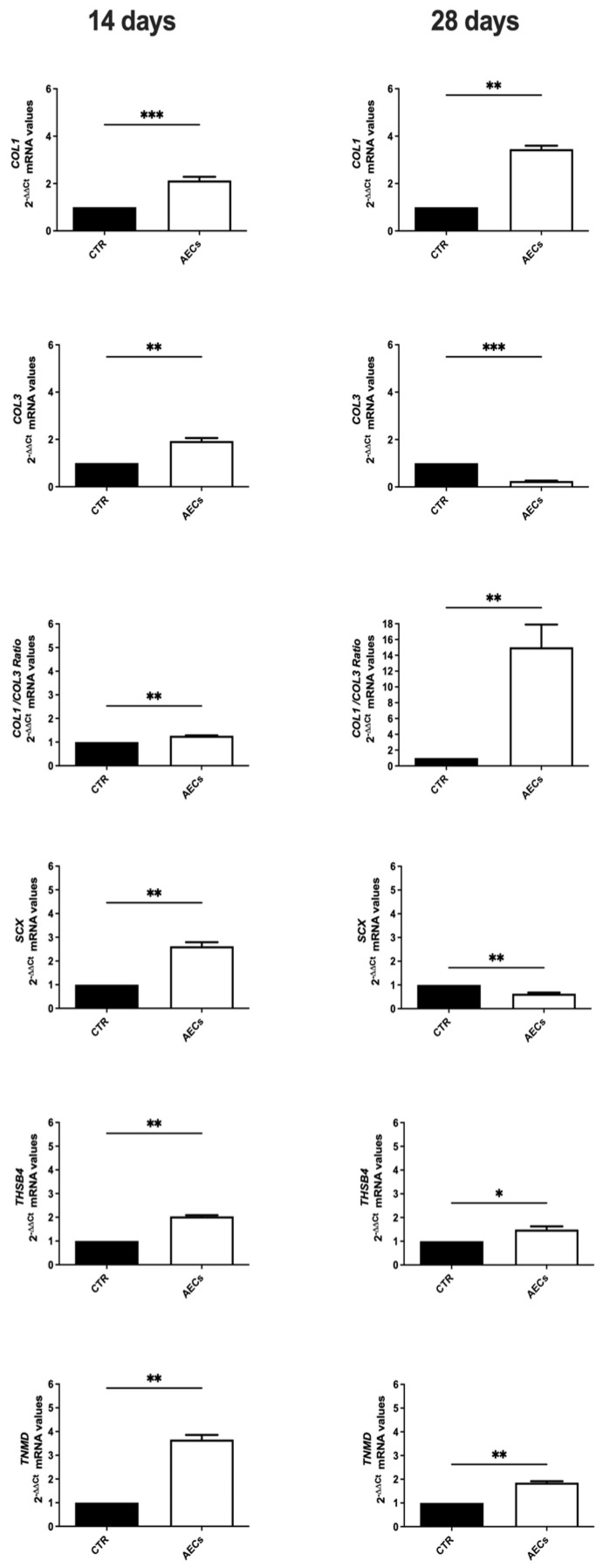
Gene expression profiles of COL1, COL3, SCX, TNMD, and THSB4 in CTR (black) and AEC-treated (white) tendons at 14 and 28 days after cell transplantation. Values are considered statistically significant for * *p* < 0.05 ** *p* < 0.01, *** *p* < 0.001, vs. CTR. Quantitative data are expressed as mean ± S.D.

The analysis of tendon-related gene expression shows that AEC treatment promotes its modulation, supporting more advanced tissue regeneration. Overall, COL1 transcripts were significantly higher in explants treated with AECs compared to the CTR group (*p* < 0.001 at 14 days p.i., *p* < 0.01 at 28 days p.i.) (Figure 6). Interestingly, COL3 mRNA levels were higher in AECs group at 14 days p.i. (*p* < 0.01) while being downregulated at 28 days with respect to the CTR (*p* < 0.001) (Figure 6). Thus, a higher COL1/COL3 ratio was observed at both time points in all allotransplanted tendons (*p* < 0.01) (Figure 6). Moreover, Scleraxis (SCX), a key transcription factor involved in early tenogenic differentiation, exhibited significantly higher mRNA levels in AEC-treated tendons than in CTR tendons at 14 days p.i. (*p* < 0.01), followed by a downregulation at 28 days p.i. with respect to CTR (*p* < 0.01) (Figure 6). Finally, at both time points, AEC-treated tendons showed a significantly increased expression of late tendon-related gene markers Thrombospondin 4 (THSB4) (14 days p.i.: *p* < 0.01; 28 days p.i. *p* < 0.05) and Tenomodulin (TNMD) (both 14 and 28 days p.i.: *p* < 0.01) with respect to CTR (*p* < 0.01) (Figure 6).

### 2.6. AEC Allotransplantation Enhances Tendon Microarchitecture Recovery

**Figure 7:**
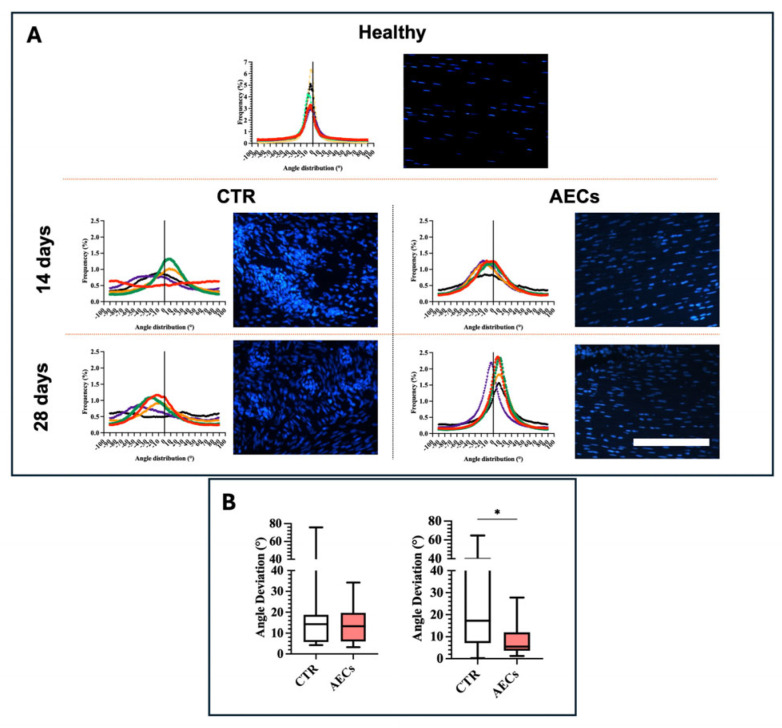
(**A**) Directionality analyses on cell orientation of the different samples at 14 and 28 days. Representative directionality curves and DAPI images of the analyzed samples within healthy, AEC-treated (AEC), and CTR tendon groups, assessed through directionality in ImageJ 1.53 k (Bethesda, MD, USA). Nuclei were stained with DAPI. (**B**) Angle deviation analysis of the CTR and AEC groups; deviation data were normalized to healthy tendons, used as a reference for the analyses. Statistically significant values were set up for * *p* < 0.05. Quantitative data are expressed as mean ±S.D. Scale bar: 200.62 μm.

Two main morphological parameters indirectly descriptive of ECM organization, i.e., cell alignment and angle deviation [14,56], were analyzed to assess the effect of AECs on tissue microarchitecture and regeneration [14,57]. Cell alignment and nuclei orientation were evaluated by analyzing the distribution of cell direction in representative images of DAPI-stained cell nuclei (Figure 7). Angle deviation was determined by calculating the difference between cell alignment directions of the CTR and AEC groups with respect to the cell alignment observed in healthy tendons (Figure 7).

Images showed a different pattern between CTR and AEC-treated tendons, both with respect to each other and to healthy tendon. In detail, a sharp Gaussian curve characterized healthy tendon cell alignment, with an average angle distribution of 4.39° ± 1.37 (Figure 7A). The CTR group showed randomly oriented nuclei characterized by a Gaussian curve with high dispersion, with an average angle distribution of 16.32°± 17.61 (Figure 7A). Similarly, the CTR Gaussian curve average at 28 days was 24.50° ± 19.81, which was consistent with the disorganized pattern observed in DAPI-stained samples (Figure 7A). Differently, AEC-treated tendons showed better cell nuclei alignment along the longitudinal axis of the tendon at both time points. While an improved Gaussian curve could be observed at 14 days, with an average distribution of 14.45° ± 9.851, at 28 days post-injury, a curve remarkably enhanced in terms of sharpness was obtained (9.04° ± 8.03) (Figure 7A), in line with evidence that AEC-based treatments promote cell nuclei alignment and thus tissue microarchitecture recovery (Figure 7A) [14,31].

As regards angle deviation, analyses showed little difference between the two experimental groups at 14 days (Figure 7B). Notably, at 28 days, CTR displayed the highest angle deviation and variability at both time points, while AEC treatment promoted a significantly reduced angle deviation of the cells at 28 days with respect to CTR (*p* < 0.05) (Figure 7B), confirming a higher degree of ECM fiber alignment. Thus, the conducted analysis confirmed the efficiency of AEC transplantation in improving tendon microarchitecture regeneration.

### 2.7. Tendon Microarchitecture Recovery Is Correlated to Neural Marker Expression

To verify the existence of a correlation between tendon morphometric parameters, cell alignment, and angle deviation and the neural markers analyzed during tendon healing, a statistical Pearson matrix analysis was performed.

**Figure 8:**
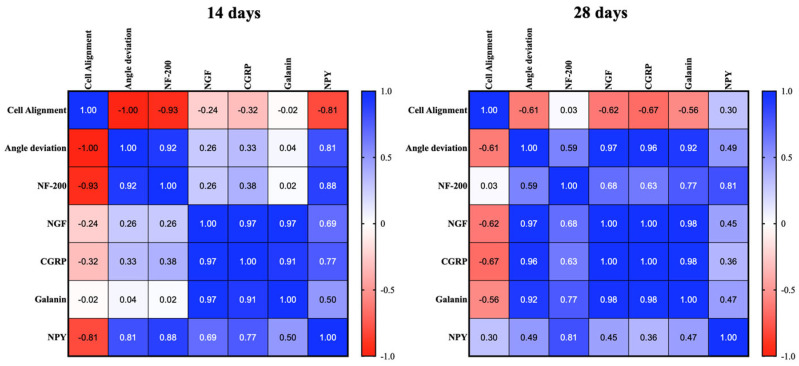
Tabular formats of the correlation matrix analysis performed amongst quantitative morphological variables (cell alignment and angle deviation) and neural markers (NF-200, NGF, NPY, CGRP, GAL) at 14 days (**left**) and 28 days (**right**) p.i. Correlational relationship representation between each quantitative analyzed metric. Colormap shows the Pearson coefficient factor distribution between 1 and −1 for positive and negative correlation, respectively.

The representative tabular format (Figure 8) highlighted that at 14 days, a strong positive correlation was found (Pearson’s coefficient > 0.91; *p* <  0.05) amongst NGF, CGRP, and Galanin (Figure 8). Interestingly, the expressions of NF-200 (Pearson’s coefficient of −0.93; *p* <  0.01) and NPY (Pearson’s coefficient of −0.81; *p* <  0.05) were negatively correlated with cell alignment. At 28 days, the data showed a strong correlation (Pearson’s coefficient > 0.98; *p* <  0.001) amongst NGF, CGRP, and Galanin. Furthermore, NGF (Pearson’s coefficient of 0.97; *p* <  0.01), CGRP (Pearson’s coefficient of 0.96; *p* <  0.01), and Galanin (Pearson’s coefficient of 0.92; *p* <  0.01) were positively correlated with angle deviation; this means that high levels of these three neural markers positively correlate with high angle deviation and vice versa.

## 3. Discussion

Recent studies highlight that the peripheral nervous system is integral to tendon homeostasis, but it is also involved during healing modulating inflammation, angiogenesis, pain, and cellular activities, collectively influencing tissue repair outcomes [23,29,54,58,59]. As regards the neuro-mediated activity in tendon healing, differently from other species (mice, rats, and humans) [15,17,23,29,53,54], the sheep, to our knowledge, has not yet been explored. This shows a gap in our understanding of the neural mechanisms leading to diverse tendon healing outcomes using an animal model of high translational value. For the first time, this study compares the spatio-temporal distribution of neural markers in a validated sheep tendon injury model of sub-optimal spontaneous tendon repair (CTR) and optimal tissue regeneration given by AEC allotransplantation [14,31,34,35,37] during the early phases of healing. In detail, in this study, as opposed to spontaneous healing (CTR), AEC-treated tissues showed a spatio-temporal modulation of NGF, NF-200, NPY, CGRP, and GAL, which statistically correlated to enhanced ECM remodeling, as demonstrated by the Pearson’s matrix analysis, highlighting these neural markers as key indicators of healing outcomes. Thus, this research highlights the neuro-modulatory role of AECs in tendon regeneration. However, the present results indicate a correlation rather than a direct causal link in the analyzed neural mechanisms.

Spontaneous tendon repair (CTR) represents the natural course of tendon repair [14,31], often resulting in the formation of scar tissue, which lacks the biomechanical properties of the native tendon [31,60,61]. Spontaneously repairing tendons reveal persistent inflammation and reduced COL1 deposition with an unfavorable COL3/COL1 ratio and disorganized collagen fibers, suggesting a delayed healing process [4,5,13,14,34,35]. Instead, treated tendons, thanks to the direct and indirect (i.e., paracrine) function of AECs, exhibit reduced inflammation, reorganization of the vascular network, and a predominance of regularly patterned COL1, indicative of advanced ECM remodeling and better biomechanical recovery [14,31,34,35,37]. Consistent with the previous literature, this study confirms that in AEC-treated tendons, an accelerated activation of tenogenic differentiation was observed at 14 days, supported by the upregulation of SCX and COL3, typically associated with early wound healing and tissue repair [14,31]. This initial response is crucial during the early stages of tendon regeneration [62]. Over time, the observed shift from COL3 towards COL1 expression aligns with the natural progression of tendon regeneration, with the latter becoming predominant as the tissue matures and gains tensile strength [63,64]. COL1/COL3 expression data are aligned with the morphological quantitative parameters of cell alignment and angle deviation, which are highly indicative of ECM organization and, thus, tendon regeneration [14,31,34,35,37]. The favorable influence of AECs on tendon healing, in fact, seems to be further confirmed by the improved distribution of somatic cell alignment and the reduced angle deviation, which reveals an improved tendon architecture in the AEC-treated group with respect to CTR. Furthermore, emerging evidence correlates the dynamics of COL1 deposition and ECM maturation to neural processes occurring in the repairing tissue [20,65]. Additionally, the upregulation of other two late tendon-related markers, THSB4 and TNMD, supported an enhanced tendon tissue maturation and ECM organization. These findings are consistent with previous evidence demonstrating that AEC transplantation significantly enhances tendon biomechanical recovery, leading to improved maximum failure load and stiffness at 28 days post-injury compared to spontaneously repairing tendons [31].

AEC allotransplantation also affected the spatio-temporal expression pattern of the neural markers analyzed in this study. Of note, AECs were characterized in terms of neural markers prior to transplantation. The results show that NPY, NGF, CGRP, and GAL are constitutively expressed in cultured AECs (P0 and P3). Furthermore, the obtained results demonstrate that this stem cell source secretes NGF in vitro at both passages. Particularly, this result is consistent with previous studies demonstrating that AECs secrete the neurotrophins NGF, BDNF, and NT-3 [47,66]. These findings are new for ovine AECs, but not for human AECs (hAECs) [67], as already demonstrated by Miki et al. [42,43,46], nor for mesenchymal stem cells (MSCs) following induced in vitro differentiation towards neurogenic lineage [68,69]. These observations imply that AECs across species might retain some neural differentiation potential [39,40,42,44], like other pluripotent stem cells [40,45,46], and may thus possess intrinsic properties that favor neural repair pathways.

The examination of the explanted tendons confirmed the survival of PKH26-labeled AECs in the tendon matrix up to 28 days post-transplantation. Furthermore, surviving AECs within the injured environment actively contributed to the healing process. Labeled AECs within the transplanted tendons, in fact, co-localized with NPY, NGF, CGRP, and GAL, likely influencing tendon regeneration. Interestingly, while NF-200 did not co-localize with PHK 26-labeled cells, neurofilaments were found surrounding clusters of transplanted cells. The high in vitro levels of AEC-secreted NGF suggest a potential to stimulate sensory nerve fibers sprouting in vivo, consistent with evidence that exogenous NGF in vivo promotes nerve ending development [70], supporting the hypothesis of active crosstalk between transplanted cells and host tissue.

Within the host tissues, a differential expression of neural markers was observed between CTR and AEC-transplanted tendons, exhibiting time-dependent patterns suggesting their potential role in controlling nerve ingrowth and nerve-mediated healing. In healthy tendons, the baseline expression of neural markers was localized in the paratenon and endotenon but was absent in the tendon proper, as reported in other species [22,71,72]. Following injury, nerve ingrowth in CTR tendons, assessed via NF-200 expression (a marker of mature nerve fibers [20]), peaked at 14 days p.i., extending toward the center of the lesion. By 28 days, nerve endings, though reduced, remained distributed across all tendon areas, indicating a persistent neural presence. In contrast, AEC-treated tendons showed NF-200 expression confined to the paratenon and to the endotenon at 28 days p.i., suggesting nerve fiber retraction and potential neural remodeling [20]. Nerve ingrowth and outgrowth play crucial roles in tendon healing [20,29]. Spontaneous repair involves extensive nerve ingrowth in the tendon proper and a time-dependent emergence of sensory, autonomic, and glutamatergic neuro-mediators that regulate inflammation and healing [20,53]. In tendon injury, excessive and prolonged nerve ingrowth may contribute to inflammatory, painful, and hypertrophic tissue reactions [20,29]. During the late stages of tendon healing, a retraction of innervation from the tendon proper is expected to occur, leading to a favorable healing response [15,73]. Thus, if not properly modulated, persistent sensory ingrowth can lead to chronic tendon injury [54,72].

In this context, NGF expression not only plays a critical role in nerve ingrowth and survival but might also correlate to tissue remodeling through the regulation of ECM adhesion molecules [74]. NGF exhibited a more widespread localization across all tendon areas at 14 days in both groups, with higher expression levels in the AEC-treated tendons than in CTR. This widespread distribution persisted and increased over time in the CTR group at 28 days, whereas in AEC-treated tendons, NGF was limited to the endotenon and close to the paratenon, and its expression was significantly lower with respect to CTR. This result suggests a restricted and more specific area of nerve activity or a shift towards the stabilization of nerve ingrowth as part of the regenerative process in treated tendons. On the contrary, the persistence in CTR tendons could be indicative of chronic inflammation and pain, associated with reduced tissue reorganization [75], as suggested by the correlation found between NGF and angle deviation data at 28 days p.i.

Similarly, for the neural markers NPY, CGRP, and GAL, AEC transplantation resulted in distinct expression patterns compared to the CTR group. NPY is involved in modulating vascular responses and inflammatory processes [20,29,72]. In the CTR tendons, NPY distribution was more evident in central areas over time, mainly associated with perivascular structures, suggesting a role in sustaining vasomotor activity and potentially chronic inflammation [17,28,76]. Conversely, in AEC-treated tendons, a more diffuse expression pattern of NPY was observed, with a slight increased fluorescence intensity over time, indicating a more controlled neural and vascular response [15,17,19]. In human Achilles tendons, NPY receptors are strongly expressed in tenocytes and blood vessels walls [28]. Ackerman et al. correlate increased hypoxia resulting from NPY-induced vasoconstriction to the switch from COL3 to COL1 expression, occurring during correct tendon healing [14,20], as further supported by experimental data regarding the favorable COL1/COL3 ratio found at 28 days.

CGRP and GAL, neuropeptides involved in pain modulation, inflammation, and vascular dynamics [20,23], also displayed distinct patterns. In the CTR tendons, their levels remained stable over time, with intense localization around blood vessels. In contrast, AEC-treated tendons exhibited a decrease in CGRP and GAL expression from 14 to 28 days p.i., which became confined mainly to the endotenon, mirroring their distribution in healthy tendons [20,53]. GAL’s downregulation from 14 to 28 days in treated tendons might correlate to the decrease in inflammation in AEC-based treatments [77]. Similarly, CGRP, which exerts anti-inflammatory activity, influencing vasodilation and ECM homeostasis [28,78,79], was linked to the substitution of COL3 by COL1 [78]; indeed, at 14 days p.i., corresponding to the shift in COL expression, CGRP was significantly higher than that in CTR. However, CGRP and GAL in AEC-treated tendons at 28 days p.i. may indicate that AECs attenuate neural inflammatory signals, thereby reducing pain and inflammation and promoting a regression of vascularization. The reduced CGRP and GAL expression in AEC-treated tendons, along with limited vascular ingrowth, suggests that AECs might inhibit excessive neovascularization, a feature linked to chronic tendon disorders and overall poor healing [14,79]. This hypothesis is further supported by the improvement in tissue architecture observed in AEC-treated tendons, indicating enhanced remodeling and potentially reduced fibrosis [14].

In this study, interestingly, several host tissue cells were positive in the cytoplasm for all analyzed neural markers. This novel finding challenges conventional assumptions regarding neural interactions with the tendon cells. Notably, in healthy tendons, these neural markers were not expresses within the cytoplasm of the cells within the tendon proper, indicating that during homeostasis, they are not engaged in neurobiological mediating mechanisms. It can be hypothesized that these cells can be referred to as tenocytes, as recently demonstrated in vivo by Faydaver et al. [53] in mice and as shown in previous in vitro or in vivo studies for the single positivity of tenocytes for NF200 [80,81], NGF [82], CGRP [29], and NPY [83]. This finding surpasses the traditional view of tendon cells as passive responders to mechanical stimuli, recognizing the role of both the recipients and generators of neural marker signals in influencing tendon healing [23].

The combined pattern of neural markers, including NGF and NF-200, NPY, CGRP, and GAL, provides a more comprehensive prediction of a favorable or unfavorable healing response. Pearson’s correlation analysis showed a relevant positive link between angle deviation, NF-200, and NPY at 14 days, consistent with the notion that while nerve ingrowth is indeed necessary in the early phases of tendon healing, the extent of ingrowth and its dysregulation are determinant for impaired tendon healing. Notably, nerve terminals associated with NPY may be involved not only in the regulation of vasomotor activity but also in modulating and counteracting hyperalgesia in acute inflammatory states [84], consistent with the correlation initially observed at 14 but not at 28 days p.i. On the other hand, NGF, CGRP, and GAL appear to be crucial neural markers for healing outcomes in injured tendons [16,20]. Indeed, there was a strong positive correlation between these neural markers at both 14 and 28 days p.i. The existing literature underlines the role of NGF in controlling the activity of peripheral sensory innervation and its ability to regulate the expression of neuropeptides CGRP and GAL [85,86], as supported by the positive correlation found at all time points among NGF, CGRP, and GAL expression. Moreover, the positive correlation between NGF, CGRP, GAL, and angle deviation suggests that the levels of these markers could be predictive for a successful or unsuccessful tendon healing outcome. In particular, in CTR (spontaneously healing tendons), high levels of NGF, CGRP, and GAL, as shown by MFI analysis, probably contributed to chronic inflammation, a consequent disorganized ECM—as indicated by the high angle deviation—and, ultimately, to fibrotic scar formation. In contrast, in AEC-treated tendons, decreased levels of NGF, CGRP, and GAL (i.e., MFI) are linked to proper ECM remodeling (i.e., low angle deviation), supporting tendon regeneration.

Based on the findings of this study, neural markers emerge as potential predictive indicators for tendon regeneration, particularly in the context of comparing AEC-treated pro-regenerative tendons to the spontaneous repairing CTR group. The differential expression patterns of these markers suggest that they may act in a cooperative fashion, influencing nerve ingrowth and outgrowth, inflammatory responses, and tissue remodeling [20]. Indeed, while this study proves the existence of a statistically significant correlation between some of the analyzed neural markers and the structural reorganization of the tendon ECM, knowledge of the detailed mechanisms behind it is still elusive. Future studies should explore the molecular mechanisms underlying AEC-mediated modulation of neural markers and their effects on healing to optimize tendon repair strategies. While in this study, NGF was selected as a representative neurotrophic factor due to its well-documented role in nerve ingrowth and tissue remodeling, a broader secretome analysis of the secretomic profile of AECs will be needed to help elucidate these mechanisms. Understanding how these cells interact with the native tendon cells and the surrounding microenvironment will also be crucial for the optimization of their therapeutic potential. Exploring strategies to fine-tune neural signaling—whether through cell-based approaches or pharmacological interventions—could further enhance tendon repair outcomes and minimize long-term complications. Additionally, future studies will need to assess the long-term involvement of neural elements in the later stages of tendon healing. Of note, this study has demonstrated that AEC applications, through their neural marker expression and influence on collagen remodeling, represent a promising avenue for predicting tendon regeneration.

## 4. Conclusions

This research provides insights into the neurobiological mechanisms underlying early tendon healing and lays the groundwork for the identification of neural marker expression patterns that could predict successful tendon regeneration.

Results show that AEC transplantation, differently to CTR, modulates NF-200, NGF, NPY, CGRP, and GAL, promoting controlled neural response, reducing aberrant nerve ingrowth, and enhancing tendon regeneration. Unlike CTR tendons, where neural markers localize near large blood vessels, at 28 days p.i., AECs refine neural modulation within the endotenon. Indeed, the positive correlation between NGF, CGRP, GAL, and cell angle deviation suggests that the levels of these markers could be predictive for a successful tendon healing outcome. A time-dependent downregulation of NGF, CGRP, and GAL in AEC-treated tendon correlates with successful regeneration and tissue organization.

These findings highlight neural markers as potential predictors of healing outcomes and suggest neural modulation as the key for improved tendon healing through targeted therapies.

## 5. Materials and Methods

### 5.1. Ethics Statement

Ovine AECs (AECs) from the amniotic membranes of pregnant slaughtered animals were obtained at a local sheep slaughterhouse. No ethics statements are required for such samples. Sheep Achilles tendon experiments were conducted in compliance with the Italian National Laws (Legislative Decree n.26/2014) and the European Community Council Directive 2010/63/EU on the Protection of Animals used for Scientific Purposes, upon approval by the Ministry of Health (approval ID 1205/2015-PR of 18 November 2015). All animals were reared and cared for according to E.D. 2010/63/UE upon performing the experimental tendon lesions. Animals were isolated for 2 weeks to check their general health status. Surgical procedures were carried out in an authorized veterinary hospital.

### 5.2. AEC Isolation and Culture

AECs were isolated from ovine amniotic membranes (AMs) from three different fetuses at ∼2–3 months of pregnancy, as described in Barboni et al. [33]. Cells were seeded at 3 × 10^3^/cm^2^ and cultured for three consecutive passages (P3) in standard medium (SM) consisting of α-MEM supplemented with 10% FBS (Gibco), 1 mL/100 mL L-glutamine, antibiotics and an antimycotic (penicillin G sodium 100 U/mL, streptomycin 100 mg/mL, amphotericin B 0.25 mg/mL; Gibco, Invitrogen, Carlsbad, CA, USA) [33,34].

Flow cytometry analysis of AECs was carried out as previously established to confirm their positivity for stemness markers (TERT, SOX2, OCT4, and NANOG [14,31,34,35,57] and surface adhesion molecules (CD29, CD49f and CD166) [14,31,34,35,57] and negativity for markers of tenogenic differentiation [14,33,34,35,57], tenomodulin (TNMD), type I collagen (COL1), and scleraxis (SCX), as well as negativity for hematopoietic markers (CD14, CD58, CD31 e CD45) [14,33,34,35,57].

From the same pools of AECs, freshly isolated (P0) and P3 cells were also used for the in vitro characterization of the expression of neural markers and, only at P3, for allotransplantation protocol in injured tendons.

Before cell transplantation, P3 AECs were subjected to cell membrane labeling with PKH26 according to the manufacturer’s instructions (S-MINI26-1KT, Sigma Aldrich, St. Louis, MO, USA). PKH26 is a red fluorescent lipophilic dye that binds stably to lipid regions of the cell membrane. Briefly, AECs were re-suspended in 1 mL of Diluent C and then 1 mL of Dye Solution containing 4 µL of PKH26 was added. The cell-containing suspension was incubated for 5 min at room temperature, mixing periodically. The staining process was stopped by adding 2 mL of PBS/BSA 1% for 1 min and, in the end, centrifuging at 400× *g* for 10 min. Cells were resuspended and counted to obtain 1 × 10^7^ PKH26-labeled vital cells to be used for transplantation. Prior to transplantation, cells were pre-conditioned overnight in homologous sera derived from the animals included in the study [37]. Aliquots of 1 × 10^7^ P3 AECs were stored in liquid nitrogen in cryovials until their transplantation.

### 5.3. Immunocytochemistry (ICC) Analysis for the Assessment of Neural Markers in AECs Before Transplantation

Prior to allotransplantation in the injured tendons, expression of the neural markers NPY, NGF, CGRP, and GAL was assessed in AECs at both P0 and P3 (Table 1). Briefly, cells were seeded on coverslip glasses in 6-well plates at a density of 80,000 cells/well in 2 mL SM. After 24 h, the medium was removed and cells were washed three times in phosphate-buffered saline (PBS); cells were then fixed in 4% paraformaldehyde/PBS (Thermo Fisher, Waltham, MA, USA) for 15 min. Upon fixation, all samples were washed with 0.05% Tween 20 1% BSA/PBS. For the blocking step, samples were incubated at room temperature (RT) in 0.05% Tween 20 1% BSA/PBS for one hour. Afterwards, each sample was incubated overnight in 1:500 of the corresponding secondary antibody (Table 1) in PBS/1% bovine serum albumin (BSA). Samples were then washed again with 0.05% Tween 20 1% BSA/PBS and then incubated for one hour with 1:500 Alexa Fluor 488 (Invitrogen Ltd., Paisley, UK) secondary antibody in 0.05% Tween 20 1% BSA/PBS. Afterwards, samples were washed three times in PBS and nuclei were stained with 1:100 4′,6-diamidino-2-phenylindole (DAPI, D1306, Sigma-Aldrich, St. Louis, MO, USA). Finally, samples were washed in PBS and, subsequently, distilled water and were air-dried prior to mounting on microscopy slides.

For each replicate, a negative reaction control was included by omitting the primary antibody. The negative control was always negative.

#### Immunocytochemistry Morphometric Analysis

As regards the ICC morphometric analysis of neural markers in AECs prior to transplantation, a Nikon A1r laser confocal scanning microscope was used, using Plan Apo λ 20× objectives (numerical aperture 0.75, zoom 1.00×, refractive index 1.0, and pinhole size 42.1, 35.8 and 26.8 µm, respectively), to capture random images from each sample. Imaging was performed using the software package NIS-Elements 4.40 (Nikon Corporation, Tokyo, Japan). For each acquired image, a manual cell count was performed on a minimum of 100 cells for each replicate to calculate the positive cell ratio for each sample. Positivity for all tested neural markers was evaluated in each image captured using the ImageJ 1.53 k software (NIH, Bethesda, MD, USA) and its Cell Counter plug in. Results are expressed as percentages of positive cells over the counted cells in each analyzed sample [14].

### 5.4. NGF Analysis of AEC-Derived Conditioned Medium Content

AECs’ paracrine activity in terms of NGF release was evaluated in vitro at P0 and P3. After reaching the passage, cells were cultured for 48 h, and afterwards, both cell passages were cultured in serum-free media for 24 h. The resulting CMs were collected, centrifuged to eliminate cell debris, and processed for NGF content analysis using the Sheep Nerve Growth Factor ELISA Kit (MBS736304, MyBioSource, San Diego, CA, USA). Samples obtained from AECs at P0 and P3 passages were added to microtiter plate wells with a horseradish peroxidase (HRP)-conjugated antibody and processed according to the manufacturer’s instructions. The optical density of each well was determined by using a microplate reader set to 450 nm.

### 5.5. Adult Ovine Achilles Tendon Injury Model

Twenty adult (∼2 years) male sheep were bred under controlled conditions. Surgical procedures were carried out as established in previous studies [14,31]. Anesthetization was induced immediately prior to surgery via the administration of xylazine IM (Rompun^®^ 0.2 mg/kg; Bayer HealthCare, Leverkusen, Germany) and tiletamine-zolazepam IV (Zoletil 100 0.2 mg/Kg; VIRBAC S.r.l., Milan, Italy). Once intubated, sheep were kept under general anesthesia through continuous inhalation of 2.5% Halothane^®^ (Merial Italia S.p.A., Milan, Italy) in an oxygen mixture. A 3 cm skin incision was performed on the left pelvic limb of each animal, starting at 4 cm proximal to the tuber calcis; this was performed while keeping both tarsi under flexion. For each animal, the flexor digitorum superficialis tendon (the medial and most prominent component of Achille’s tendon) was identified, and a 5 mm diameter full-thickness hole was made; the procedure was carried out only on the left tendon. The surgically induced defect in the control (CTR) groups was sealed with fibrin glue alone (60 µL, 1:1, *v*/*v*; Tissucol/DMEM; Baxter S.p.A., Illinois, IL, USA); instead, tendon defects in the treatment groups were filled with 1 × 10^7^ AECs (isolated and cultured as previously described) prestained with PKH26 dye and then sealed with fibrin glue. Five animals were employed for each time point (14 and 28 days) in all experimental groups (treated and CTR). Skin suture was performed after the closure of the paratenon and fascia. Post-surgery, animals were quarantined in an isolated pen until euthanasia. The surgical procedures and cell transplantation did not result in any observable discomfort in the animals, which fully recovered normal movement immediately after anesthesia. Animals were euthanized either at 14 or 28 days post-surgery by thiopental and embutramide overdose (Pentothal Sodium-Intervet; Tanax^®^-Intervet, Aprilia, Italy) [14,31].

Tendons were isolated and explanted under sterile conditions; explants were then cut at least 5 mm from the lesion site, transversally, in smaller pieces of approximately 2 cm^3^ in size. The central portion of contralateral non-injured tendons was also collected as healthy control. All samples were then cryopreserved in liquid nitrogen until further processing.

### 5.6. Histological Assessment of Tissue Microarchitecture

Histological analysis was performed following a previously validated protocol [56]. Tendon explants were fixed in 4% paraformaldehyde/phosphate-buffered saline (PBS) for 1 h. Samples were then dehydrated using a range of alcoholic solutions at increasing concentrations, from 70 to 100%, and embedded in paraffin wax. For histological analysis, samples were sliced to a thickness of 7 μm using a microtome and collected on poly-lysine adhesion microscope slides. Samples were then immersed in xylene for 10 min to remove paraffin, followed by a series of alcohol solutions at decreasing concentrations (from 100 to 70%) for 30 s and distilled water to rehydrate the sections. Subsequently, hematoxylin/eosin (H-E) staining was performed for a standard histological observation [87].

### 5.7. Immunohistochemical Assessment of Neural Marker Expression in Tendon Explants

Following the allotransplantation, immunohistochemistry (IHC) was performed on tendon sections obtained from the cryopreserved explants to assess the expression and localization pattern of NPY (autonomic signaling), CGRP (sensory signaling), and Gal (opioid signaling). NGF was evaluated as a general neurotrophic marker, and Neurofilament (NF-200) was used to determine the presence of nerve endings (Table 1).

Sections cut at a thickness of 12 μm were washed twice in PBS and were then fixed for 10 min in 4% paraformaldehyde/PBS. After fixation, sections were washed three times in PBS and then incubated with the respective blocking agent for one hour, followed by overnight incubation with the primary antibody (Table 1). After overnight incubation, slides were washed three times in PBS and incubated with secondary Antibody AlexaFluor 488 anti-rabbit (Invitrogen Ltd., Paisley, UK) 1:250 in PBS/1% bovine serum albumin (BSA) for one hour. Slides were then washed again three times in PBS, and nuclei were stained using 1:100 DAPI (D1306, Sigma-Aldrich, St. Louis, MO, USA) in PBS.

For each replicate, a negative reaction control was included by omitting the primary antibody. The negative control was always negative.

#### Immunohistochemistry Morphometric Analysis

All tissue samples were observed using the above-mentioned Nikon A1r laser confocal microscope setup; imaging and analysis was performed using the software package NIS-Elements 4.40 (Nikon Corporation, Tokyo, Japan).

The identification and localization of PKH26-labeled AECs (λexcitation = 551 nm, λemission = 567 nm) was also carried out using said Nikon setup. The DAPI channel (λexcitation = 359 nm, λemission = 461 nm), FITC channel (λexc = 488 nm; λem = 525 nm), and TRITC channel (λexc = 544 nm; λem = 570 nm) were used in this study [77]. Analyses were performed at 20× magnification, capturing four images from three contiguous areas starting from the outer area of the tendon, closer to the paratenon (area 1), and proceeding throughout the repairing zones for three consecutive areas (areas 2 and 3), as adapted from a previously published report [14]. The extension of each field analyzed was ~642 μm; thus, the total surface analyzed within each area was ~2568 μm. The acquisition settings for all images were initially calibrated using the negative control (a sample processed without the primary antibody). Once the optimal settings were established, all images were acquired using the same parameters for each fluorescence channel to ensure consistency.

Colocalization analysis was performed by acquiring images in different channels corresponding to each dye, generating distinct fluorescent images, which were then superimposed: overlapping signals indicated potential co-localization. Then, on the acquired images, the Region of Interest (ROI) function in NIS-Elements ER 6.10 was used to quantify the spatial overlap between selected markers. Specifically, colocalization was assessed using both Pearson’s correlation coefficient (PCC) and Manders’ overlap coefficient (MOC), which provide complementary insights into signal distribution [88]. Pearson’s coefficient measures the linear correlation between the fluorescence intensities of two channels, with values ranging from −1 (perfect inverse correlation) to +1 (perfect correlation), indicating the degree of colocalization [88]. Manders’ coefficients (M1 and M2) quantify the proportion of signal from one channel overlapping with the other, offering a more intensity-dependent evaluation, with values ranging from 0 to 1 (with 0.5 meaning that 50% of the selected channels overlap) [88]. ROIs were carefully selected within the tendon lesion site to ensure accurate analysis, minimizing background noise and ensuring biological relevance. This approach allowed us to precisely evaluate the spatial interaction of transplanted cells and neural markers within the regenerating tissue [31,37,88].

Following previously validated protocols [14,56], the fluorescence intensities of all samples subjected to immunostaining for all neural markers considered were evaluated using the RGB Profiler plugin within the ImageJ 1.53 k software (NIH, Bethesda, MD, USA). Each image underwent processing through this plugin, generating red, green and blue profile plots on a unified graph for each image. The results were expressed as mean fluorescence intensity (MFI), providing a visual representation of both the minimum and maximum fluorescence values. To account for background fluorescence, the average intensity value of the negative control was subtracted from all measured values. Finally, the corrected fluorescence intensities were normalized to the average intensity of the experimental CTR group.

Moreover, quantitative analyses were carried on immunohistochemical images to measure cell alignment and angle deviation, tendon morphometric parameters that indirectly indicate ECM organization [14,56], using the directionality plugin in ImageJ 1.53 k (NIH, Bethesda, MD, USA) software to assess the extent of tissue reorganization, as detailed in a previously published report [14,56]. Cell alignment analyses were performed with an Axioscop 2plus epifluorescence microscope (Zeiss, Oberkochen, Germany) equipped with a cooled color charge-coupled device camera (CCD; Axiovision Cam, Zeiss) interfaced with an interactive and automatic image analyzer (Axiovision, Zeiss) at 20× magnification by acquiring 5 randomly selected fields from each area, starting from area 1 and continuing throughout the repairing areas (areas 2 and 3). The cell alignment and nuclei orientation assessment values reflect the tallest peak observed at the center of the Gaussian (mean) and the Gaussian standard deviation (S.D.) (dispersion). Angle deviation was determined by calculating the difference between the cell alignment directions of the CTR and AEC groups compared to healthy tendons; values were standardized to healthy samples [14]. All analyses were conducted by a single, blinded examiner to ensure the reliability of results.

### 5.8. Collagen Type 1 and Type 3 Genes Expression Profiling

#### 5.8.1. Laser Capture Microdissection (LCM)

Cryosections of 12 μm in thickness were briefly air-dried on uncoated glass slides and washed with 70% ethanol. The sections were kept on dry ice at −80 °C until they were needed for LCM. Upon performing the procedure, the sections were fixed in 70% ethanol for 10 s and stained with H&E. LCM was carried out using a laser capture microdissection device (MMI Cellcut, Eching, Germany) with the following settings: laser power 50 mW, pulse duration 50 ms, spot diameter 10 μm. The area of interest was identified at ×640 magnification under a light microscope. The microdissected injured area including the implantation site was moved to a separate support prior to total RNA extraction. The portion of healthy tendon directly contiguous to the injured area was also microdissected to collect it as a healthy internal control of each animal for the investigation. Total RNA from all microdissected sections was extracted for use in RT-qPCR.

#### 5.8.2. Total RNA Extraction and RT-qPCR

The expression of tendon-related gene markers (Table 2) was evaluated via RT-qPCR on AEC-treated and CTR microdissected cryosections of tendon explants [14,31]. For each animal, healthy tendons from the contiguous injured area and contralateral healthy tendon were used to define the baseline gene expressions. TriReagent (Sigma Aldrich, St. Louis, MO, USA) was used for total RNA extraction from cryosections of both microdissected defective tendon areas (*n* = 5 for each group/time) and from microdissected healthy tendon contiguous to the injured area (*n* = 5 for each group/time) [34,37], following the manufacturer’s instruction. Following the evaluation of RNA integrity and DNase I digestion, reverse transcription to cDNA was performed using 1 μg of total RNA of each sample. Two-step cycling RT-qPCR was performed for each sample, from each animal separately, by using specific gene primers and each gene value was normalized to the endogenous housekeeping gene GAPDH (Table 2). For each treated animal, the intra-relative expression of each target gene in the injured tendon was calculated by the comparative Ct (∆∆Ct) method against the contiguous and contralateral healthy tendon [89]. To compare gene expression levels between treatment groups, target gene values were expressed as fold change over CTR tendon, hereby set as 1. For statistical purposes, the mean of three independent experiments/animal was considered.

### 5.9. Statistical Analysis

Quantitative data from AEC in vitro characterization were assessed for their normality distribution with the D’Agostino Pearson test, and they then were compared as mean ± S.D. using a one-way ANOVA followed by the Tukey post hoc test (ImageJ 9, GraphPad Software, San Diego, CA, USA) on at least three samples for each biological replicate (*n* = 3 animals). Significant values were considered for at least *p* < 0.05.

As regards the in vitro assessment of neural markers, experiments were conducted in replicate for each pool of cells from a different fetus. The normality of the data was assessed in GraphPad Prism through the Shapiro–Wilk and Kolmogorov–Smirnov tests, followed by statistical analysis via the unpaired T test. Again, statistical significance was assumed for *p* < 0.05. All results are expressed as mean ± standard deviation (S.D.)

Tendon explants for all time points in both conditions were isolated from five different animals each (*n* = 5 biological replicates). For each animal, all experiments were conducted in triplicate. Data analysis was performed using GraphPad Prism 9.5.1 (GraphPad Software, San Diego, CA, USA). Quantitative and semi-quantitative data from all and in vivo experiments were assessed for normality through the Shapiro–Wilk and Kolmogorov–Smirnov tests. For the analysis of the expression of neural markers, the Welch ANOVA was used, followed by the Tamhane T2 test for multiple comparisons. Data were considered statistically significant when *p* < 0.05. Results were expressed as mean ± standard deviation (S.D.). For the analysis of ECM- and tendon-related gene expression, the unpaired *t*-test was used for the analysis of data for each gene at each time point. Moreover, Pearson’s correlation analysis was performed on the data obtained from the samples at both time points to assess and quantify the correlations between the tendon morphometric parameters of cell alignment and angle deviation and all neural markers. Confidence intervals for Pearson’s correlation coefficients were calculated using Fisher’s Z’ transformation (GraphPad Prism 9, GraphPad Software, San Diego, CA, USA).

## Figures and Tables

**Table 1:** Primary antibodies used for the immunocytochemical and immunohistochemical assessment of the expression of neural markers.

Antibodies	Dilutions	Manufacturer
NPY	ICC: 1:500 IHC: 1:500	N9528, Sigma-Aldrich, St. Louis, MO, USA, Polyclonal
NF-200	ICC: 1:500	N4142, Sigma-Aldrich, USA, Polyclonal
CGRP	ICC: 1:500 IHC: 1:100	AB1971, CHEMICON, Temecula, CA, USA, Polyclonal
GAL	ICC: 1:500 IHC: 1:400	MBS565327, MyBioSource, San Diego, CA, USA, Polyclonal
NGF	ICC: 1:500 IHC: 1:400	N6655, Sigma-Aldrich, St. Louis, MO, USA, Polyclonal

**Table 2:** Primers details used for RT-qPCR analysis. Primers were selected from previous reports a [14,57], b [14,37], c [14,34]; H.K.: housekeeping gene.

Gene	Accession No.	Forward	Reverse	Product Size (Bp)
COL1 ^a^	AF129287.1 Ovine	5′-CGTGATCTGCGACGAACTTAA-3′	5′-GTCCAGGAAGTCCAGGTTGT-3′	212
COL3 ^b,c^	AY091605.1 Ovine	5′-AAGGGCAGGGAACAACTTGAT-3	5′-GTGGGCAAACTGCACAACATT-3′	355
TNMD ^a^	NM_001099948.1	5′-TGGTGAAGACCTTCACTTTCC-3′	5′-TTAAACCCTCCCCAGCATGC-3′	352
TBSH4 ^b,c^	NM_001034728.1	5′-CCGCAGGTCTTTGACCTTCT-3′	5′-CAGGTAACGGAGGATGGCTTT-3′	231
SCXB ^a^	XM_866422.2	5′-AACAGCGTGAACACGGCTTTC-3′	5′-TTTCTCTGGTTGCTGAGGCAG-3′	299
GAPDH ^a^	AF030943.1 Ovine	5′-CCTGCACCACCAACTGCTTG-3′	5′-TTGAGCTCAGGGATGACCTTG-3′	224

Primers were selected from previous reports a [14,57], b [14,37], c [14,34]; H.K.: housekeeping gene.

## Data Availability

The datasets used and/or analyzed during the current study are available from the corresponding author on reasonable request.
